# eHAPAC: A Privacy-Supported Access Control Model for IP-Enabled Wireless Sensor Networks

**DOI:** 10.3390/s19071513

**Published:** 2019-03-28

**Authors:** Fagui Liu, Yangyu Tang, Liangming Wang

**Affiliations:** 1 School of Computer Science and Engineering, South China University of Technology, Guangzhou 510006, China; fgliu@scut.edu.cn; 2 School of Software Engineering, South China University of Technology, Guangzhou 510006, China

**Keywords:** access control, resource-constrained device, privacy-preserving, blockchain, wireless sensor network

## Abstract

The implementation of IP technology in wireless sensor networks has promoted the development of many smart scenarios. To enhance secure access in IP-enabled wireless sensor networks, access control to sensor nodes is a necessary process. However, access control currently faces two challenges, feasibility and preservation of user access privacy. In this paper, we propose eHAPAC, a novel privacy-preserving access control model for IP-enabled wireless sensor networks. The contributions of our paper include three parts. First, this paper integrates the Hidra access control protocol and APAC privacy-preserving model, addressing the issue of privacy-preserving access control in resource-constrained devices. Second, this paper proposes an enhanced Hidra protocol to implement the unlinkability of protocol message exchanges. Third, to solve the problem of third party credibility, this paper improves the group signature-based APAC model and utilizes blockchain technology to manage the storage and publication of public group signature keys. Security analysis and performance evaluation prove that our protocol is secure and effective.

## 1. Introduction

Wireless sensor networks (WSNs), as an important part of the Internet of Things (IoT), enable us to create smart environments. They are typically composed of thousands of tiny, low-cost, low-power, resourced-constrained sensors that detect environment conditions such as temperature, noise, light, or the movement of objects. With their low cost, WSNs have been widely used in military reconnaissance, industrial monitoring, medical health care and other fields [1,2].

However, there are still many problems in traditional WSNs, such as poor scalability and single point of failure issues [3,4]. Recently, in virtue of the development of solutions like the 6LoWPAN standard, the problems which block the native integration of sensors and the Internet (e.g., IPv6 header overhead, packet transmission, etc. on the IEEE 802.15.4 network) have been solved [5,6,7]. The end-to-end (E2E) communication between Internet users and sensor nodes can really be realized, which promotes the application of WSNs. However a new security crisis has been introduced for IP-enabled WSNs whereby adversaries can more easily access data on sensor nodes by using global addressing [8,9,10]. Moreover, the high frangibility of the WSN itself (e.g., its resource constrained nature) makes it a easy target for many security attacks [3,11] (e.g., hacking, data theft, remote hijacking) or a tool for hackers to launch security attacks [12] (e.g., Mira, composed of IoT devices that constituted a million-level botnets, launched a crazy DDoS attack on Krebson Security). Therefore, the access to sensor nodes must be strictly controlled in IP-enabled WSNs. A basic access control model includes three components: authentication, authorization and auditing [13]. Generally, authentication and authorization imply transmitting user identity-related information to the targeted service, which will cause another problem, namely privacy security of data access. Currently, user access behavior is a target for data collection [13], from which users’ behavior patterns and preferences can be summarized, posing a threat to users’ privacy and even property and lives [14,15,16,17,18]. Therefore, it is necessarily to implement access control in IP-enabled WSNs without revealing users’ private information.

Although there are many mature traditional access control models, the particular properties of WSNs make the implementation of those models face two main challenges: (1) Sensors are resource constrained. Sensors are subject to strict resource constraints, whether in terms of computing power, storage capacity, or transportable overhead so that many mature traditional access control models are unfeasible. (2) Privacy disclosure. User access pattern may be closely related to users’ social work, preferences and other private information. Most traditional access control methods do not consider user privacy preservation. It is possible that potential eavesdroppers can analyze the user data access records to further obtain users’ private information. 

Recently, various mechanisms have been proposed to address user privacy-preserving access control in WSNs. Those mechanisms are of two main types: one aims to hide the user identity by introducing cryptographic mechanisms, such as the group signature-based APAC privacy-preserving model [19]. In these schemes, privacy preservation relies on the difficulty of resolving some problems, so such schemes have strong privacy security, but most of them lack any consideration of feasibility in a resource-constrained scenario. The other type implements access control by introducing an absolutely trusted third party, which stores the mapping table of the user real identities and a pseudonym set, such as the enhanced Ladon protocol [20]. This way users can be authenticated and authorized by sending a pseudonym to the third party. However, this type of model fully entrusts the user privacy to third parties. Once the third party is attacked or the data is leaked, all users’ data access privacy will be threatened. Additionally, the users may come from different organizations, have different identities and exist in different forms. Because of the interest in the data provided by sensor nodes, they register against the administrator of sensor networks to make themselves the legal acquirer of sensor node data. Generally, in the aspect of privacy protection, users and the administrator of sensor networks may have conflicting interests, so there is no trust relationship among them. Blindly trusting a third party may have serious consequences, such as a third party privately selling the data access records, or handing them over to a government agency, resulting in user access trends being monitored [21,22].

According to the above problems and challenges, a privacy-preserving access control model in the IP-enabled WSN should meet the following requirements: (1) Basic access control mechanism. Authentication and authorization: ensure that only legitimate user with permissions can access the nodes. Auditing and accountability: identify misbehaviors and misbehaving users. (2) Feasibility. IETF [23] classifies constrained devices in three levels (C0~C2). The C0 class devices are the most constrained devices. Making such devices able to communicate directly with the Internet in a secure manner is the target of feasibility. (3) User data access privacy preserving. Any entity cannot associate the identity of a user with a data access record, nor can it determine whether two data access records are from the same user. 4) Resist basic network attacks. The model should be able to resist common network attacks, such as replay attacks and resource consumption attacks.

In this paper, we propose a novel user privacy supported access control model in the IP-enabled WSN, namely eHAPAC. The main contributions are listed as follows:

- We propose a privacy-enhanced Hidra protocol by integrating the original protocol with the privacy enhancement mechanisms of the enhanced Ladon protocol. The enhanced Hidra protocol supports unlinkability of protocol message exchanges and the feasibility of access control in severely resource-constrained environments.
- We propose a privacy-preserving model to implement anonymous authentication that exploits the group signature technique. Our privacy-preserving model improves the APAC privacy-enhanced access control model by designing a new key generation scheme, so as to protect the key generator from linking user authentication request messages.
- We propose a series of mechanisms to enhance security of our eHAPAC model. Blockchain is introduced into the accountability mechanism to disclose the accountability calculation procedure in order to solve disputes in our privacy-preserving model. A novel blockchain-based key publish mechanism and a novel blockchain-based message exchange mechanism are proposed to increase the flexibility of public key management and resist resource consumption attacks respectively.
- We analyze the security and efficiency of the proposed eHAPAC model, and implement the privacy-preserving model of eHAPAC. The time consumption of the proposed privacy-preserving mode is compared with the original APAC model.

The rest of this paper is organized as follows: Section 2 discusses the relevant related work. Section 3 describes the problem formulation. Section 4 provides the detail of our model. Section 5 and Section 6 conduct security and performance analysis respectively. Finally, Section 7 shows our conclusion.

## 2. Related Work

In recent years, the security in WSNs has received great attention. Access control technology is seen as a significant security mechanism development in WSNs. Uriarte et al. [24] analyzed some traditional access control models and some current new access control models designed for resource-constrained devices (RCDs), finding that the former are not feasible in all RCDs, and the latter can only be implemented for class C2 RCDs. The authors then proposed a new access control protocol called Hidra. This protocol is based on the Ladon access control protocol, the enhanced version of the Kerberos authentication protocol. A comparison of Hidra, Ladon and Kerberos is given in Table 1. The Hidra and the Ladon protocols improve Kerberos by integrating an authorization mechanism, and Hidra protocol implements dynamic policy configuration and accurate audits based on Ladon. The authors evaluated Hidra on three indicators of power consumption, memory and response time, providing results demonstrating the availability of the protocol on class C0 RCDs. However, Hidra does not pay any attention to user privacy security.

The schemes discussed in [20,25] are two privacy-preserving access control schemes that enhance the Kerberos and the Ladon methods mentioned above by adding privacy support. The PrivaKERB [25] user privacy framework for Kerberos provides user privacy protection by integrating the original Kerberos protocol with a dynamic pseudonym mechanism and regenerating a Ticket Granting Ticket (TGT) mechanism. Reference [20] upgrades the PrivaKERB framework based on the Ladon protocol. However, the main weakness of the two schemes is the fact the user privacy security relies on a third party key distribution center (KDC). The mapping of the user real identity and pseudonym set is stored on the KDC, so the KDC can fully grasp the user access traces. Once the KDC is attacked and the data is leaked, the privacy of all users will be exposed. In addition to this, neither of the two frameworks considers resisting resource consumption attacks. 

Digital signature techniques in cryptography are progressively being applied to privacy-preserving access control. Zhang et al. [26] payed attention to this research area previously. They proposed a ${\mathrm{DP}}^{2}\mathrm{AC}$ protocol that uses blind signatures in token generation to ensure tokens can be publicly validated yet unlinkable to user identities. However, it is not fine-grained in that any anonymous user has exactly the same privilege to access the nodes. Subsequently, He et al. [27] and Han et al. [28] designed access control schemes based on ring signatures to achieve privacy-preservation. In a ring signature scheme, nobody can revoke the anonymity of the actual signer in any case [29]. Failure to track down misbehaving users is the fatal defect in these ring signature schemes. The CLSC-based anonymous access control scheme proposed by Li et al. [30] aims to protect other third parties except for the user himself and controller from knowing the user identities. This scheme uses an identity-based signature mechanism, which can easily expose the user’s identity. He et al. [19] proposed a group signature-based privacy-enhanced access control scheme. The scheme divides the user identity and private open-key (which is used to open a group signature to reveal a signer’s certificate) into two parts, which are saved by some legal authority and the WSN owners, respectively. This scheme may cause two problems: on the one hand, although the legal authority can’t know the user real identity corresponding to the signer’s certificate, it can easily link the request messages of the same user. On the other hand, in order to avoid key leakage, the accountability process can’t be above board, which is easily causes disputes. The schemes previously mentioned in [19,26,27] suffer from a serious limitation in that they fail to consider feasibility in strictly constrained devices. For example, schemes [19,27] store access control lists on sensor nodes and perform signature verification and authorization locally on sensor nodes, which is almost impossible for C0 devices with severe resource constraints.

After summarizing the related works, we can see that none of the current research work meets the requirements mentioned in Section 1. Some of these schemes ignore the feasibility of using them in resource-constrained environments, lack privacy support or have flaws in their privacy-preserving mechanism (e.g., blind trust in third parties). Therefore, our work mainly aims to solve the feasibility issue and support user privacy-preservation without blindly trusting third parties. For the other security issues mentioned above, such as resource consumption attacks, accountability disputes and so on, we propose novel mechanisms based on blockchain technology. Blockchain technology has emerged as the promising solution for creating a more secure IoT in the future [31]. Blockchain properties like transparency, irreversible, distribution and auditability can help the IoT resist many network attacks such as resource consumption and liability disputes [32,33,34,35].

## 3. Problem Formulation

In this section, we first describe the access control system architecture. Then, we provide the trust model, threat model considered and security goals we want to achieve.

### 3.1. Access Control System Architecture

**Figure 1:**
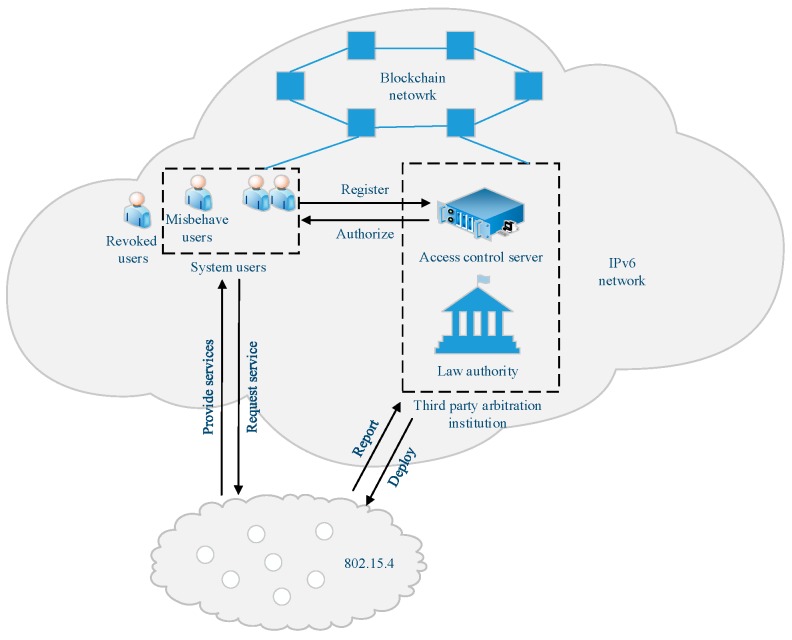
Access control system architecture.

As shown in Figure 1, there are four entities in the access control system architecture: users, a WSN based on the standard of IEEE 802.15.4, a third-party access control server (ACS) and a third-party law authority (LA):

**Users**: Users include a number of registered users who participate in the security protocol and revoked *users*. If a registered user violates the access control policy, he is called misbehaving user.

**ACS**: The ACS is an important control entity responsible for deploying the network, enforcing access control policies and tracking the specific misbehaving user.

**LA**: The LA, as an independent third party, can be a local police department. It together with the ACS to constitute a third part arbitration agency responsible for identifying the misbehaving user and resolving disputes, in an extreme case such as the server attack. Meanwhile, it also involved in the deployment of the WSN to generate parameters for accountability. 

**WSN**: The WSN consists of a group of resource-constrained sensor nodes. These sensor nodes act as the tiny information providers that are directly addressable by any Internet-connected party.

**Blockchain network**: The blockchain network is an independent network that can be one of the current public blockchains. Users, the ACS and the LA are nodes in the blockchain. For the model we designed, the blockchain is used to manage some shared parameters, resist resource consumption attacks and improve accountability mechanisms.

### 3.2. Trust and Threat Model

It is assumed that the ACS is semi-trusted, that is, it can honestly perform access control, but it is curious about user data access privacy. Moreover, the ACS is in an insecure network environment, facing the risk of data leakage caused by network attacks. It is considered that the LA is also semi-trusted, which is curious about the user privacy and may track user access trends. In addition, in the accountability process, it may collude with misbehaving users to frame others.

Our threat model considers four types of attack:

1. Attacks against user privacy: Both external adversaries and internal entities including users, the ACS, and the LA, are curious about user data access privacy. The user identities may be obtained by the way of network sniffing.
2. Replay attack: External adversaries may expect to access data without privilege by intercepting the transmitted messages and replaying them.
3. Resource consumption attack: external adversaries or internal users (registered user and misbehaving user) may generate a large number of invalid or valid request messages to consume the computing resources of the ACS.
4. Collusion between the arbitration organization and internal users: When illegal actions occur among internal users, once the arbitration organization colludes with misbehaving users, they may frame other users to prevent the identities of misbehaving users from being revealed.

### 3.3. Security Goals

In order to solve the above problems, the model in this paper aims to achieve the following security goals:

1. Anonymity and messages unlinkability: In the case of normal access to services by internal user, no one can know the real identities of requesters, including the ACS and the LA. External adversaries, internal users and the LA cannot determine whether any two data access requests originate from the same user. The ACS cannot establish a relationship between data access requests for different protocol cycle.
2. Resist replay attack: Verify the freshness of the messages.
3. Resist resource consumption attack: Guarantee that the ACS is not attacked by resource consumption to ensure availability of services.
4. Accountability: When resolving a dispute, the arbitration agency must give the identity of the real misbehaving user and provide publicly verifiable evidence.

## 4. eHAPAC Construction

In this section, we present details of the proposed eHAPAC privacy supported access control model. We first present an overview of it.

### 4.1. General Overview

**Figure 2:**
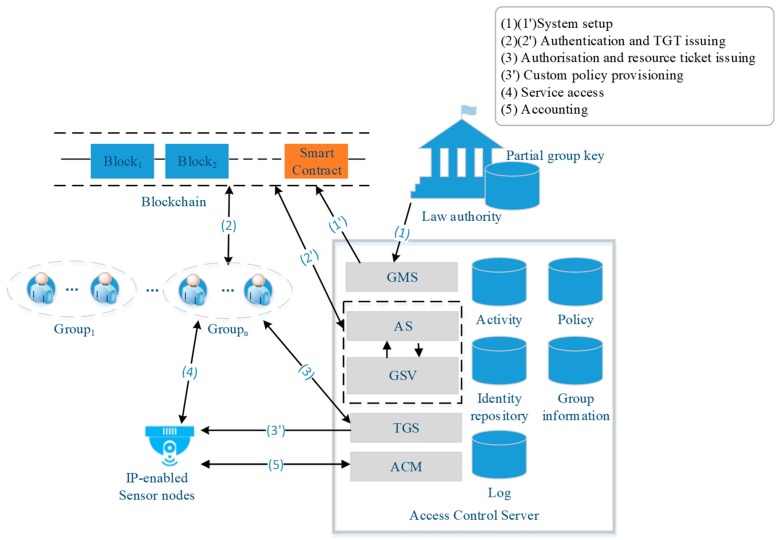
The proposed eHAPAC model.

The proposed eHAPAC model is shown in Figure 2. The privacy-preserving model of eHAPAC is based on a group signature technique. To structure the privacy-preserving model, registered users are divided into separate groups according to their different access privileges, and group users generate group signatures for authentication. The privacy-preserving model consists of six phases: system setup, new user joining, signing, verifying, user revocation and accountability. When group users access sensor nodes, the access control protocol of eHAPAC is executed, including: authentication phase, authorization phase, service access phase and the auditing phase.

The ACS is a pivotal control entity in eHAPAC. In the ACS, the group management related operations, including group key generation, user joining and user revocation, are performed by the group manager server (GMS). Authentication and issuing long-term tickets are implemented by the authentication server (AS). In the authentication phase, the AS extracts the group signatures from the request messages and submits them to group signature verifier (GSV) for verification. The ticket granting server (TGS) is responsible for authorization and issuing the service tickets. The accounting manager (ACM) performs auditing and accountability operations.

The system setup phase is to initialize eHAPAC. In this phase, the ACS and the LA cooperate to generate group keys, in which group public keys are published to the blockchain by the ACS.GMS through invoking smart contract as shown in Figure 2(1),(1′). New users acquire the corresponding group public key on the blockchain and perform user joining operations to join the network. User access to the sensor nodes is constrained by access control policies. A complete access control cycle is depicted in Figure 2. Authentication (2) (2′): The user as requester invokes the signing phase to generate a group signature for authentication to get the long-term ticket known as ticket granting ticket (TGT) from the ACS.AS. The blockchain serves as the intermediate platform for the requester and the ACS.AS to exchange messages in this phase. Authorization (3) (3′): The requester uses the TGT to apply for a service ticket. The ACS.TGS queries the policy database to determine whether the group to which the user belongs has the corresponding privileges. Then the TGS issues the service ticket and a new TGT to the legitimate user. Service access (4): The requester invokes the target service provider with the service ticket through E2E communication. The target node verifies the service ticket and establishes a secure connection with the requester if the ticket is valid. Audit (5): For each secure connection, the sensor node sends log to the ACS.ACM for audit. When subscriptions to services expire or users violate access control policy, the user vocation phase is initiated: the ACS invokes the smart contract to issue revocation transaction. In an extreme case such as network attacks, the accountability phase is to be carried out: the ACS.ACM invoke the smart contract, and cooperate with the LA to open the signature. The privacy-preserving model, access protocol and smart contract design of eHAPAC are described in detail below.

### 4.2. The Privacy-Preserving Model of EHAPAC

In this part, we present in detail the privacy-preserving scheme of our model. We choose the group signature scheme proposed by Cecile et al. [36] as an example. The eXtremely Short Group Signature Scheme (XSGS) [36] can be proved in the strong security model of Bellare.al [37]. It allows users to join and revoke dynamic and generate group signatures with shorter length than other signature schemes, thus saving storage capacity on blockchain. Table 2 presents the terms used as abbreviations and the notation in the description of the privacy-preserving model.

#### 4.2.1. System Setup

At this phase, the ACS generates partial group key and Elliptic Curve Diffie-Hellman (ECDH) public/private key pairs for each group. The ECDH public key is issued as part of the group public key, which is stored on blockchain by the ACS invoking the smart contract. Because the ACS is not able to know the real identity of the requester, so it is unable to preset the session key between each requester. The key establishment algorithm based on ECDH is used to establish the temporary session key $ESK_{U,ACS}$ between the ACS and the anonymous requester. Likewise, the LA generates the other part group key for each group.

ACS generates the partial group key:

1. Let $G_{1}$,$G_{2}$ and $G_{t}$ be three bilinear groups of prime order p with independent generators $G_{1},K\in G_{1}$, an isomorphism from $G_{2}$ to $G_{1}$ with $\psi \left(G_{2}\right)=G_{1}$ and $e:G_{1}\times G_{2}\to G_{t}$ is an efficient bilinear map.
2. Choose an RSA modulus n, and an element g of maximal order in ${\mathbb{Z}}_{n^{2}}^{*}$, keeping the factorization.
3. Generate an issue-key $\mathrm{IK}\in {\mathbb{Z}}_{P}$ which is used to issue group member certificates and user-keys. Compute $W=G_{2}^{IK}$ as the corresponding public key of IK.
4. Generate partial open-key $\xi_{1}\in_{R}{\mathbb{Z}}_{p}$, and compute its corresponding public key $H_{1}=K^{\xi_{1}}$.
5. Choose a random number $e_{ACS}\in {\mathbb{Z}}_{p}^{*}$ as ECDH private key, and compute the ECDH public key $E_{ACS}=e_{ACS}\times K$.

LA generates the partial group key:

1. Generate partial open-key $\xi_{2}$, compute the corresponding public key $H_{2}=K^{\xi_{2}}$.
2. Send $H_{2}$ to the ACS through open channel.

After this phase, the group public key gpk and group private key gsk (include issue-key IK, open-key OK and ECHD private key $e_{ACS}$) have been generated. Details are as follows:

—$gpk=\left\{G_{1},G_{2},G_{t},e,\psi ;G_{1},K,H_{1},H_{2};G_{2},W;g,n;E_{ACS}\right\}$

—$gsk=\left\{\mathrm{IK},\mathrm{OK},e_{ACS}\right\}$

—$\mathrm{OK}=\left\{\xi_{1},\xi_{2}\right\}$

#### 4.2.2. New User Join

**Figure 3:**
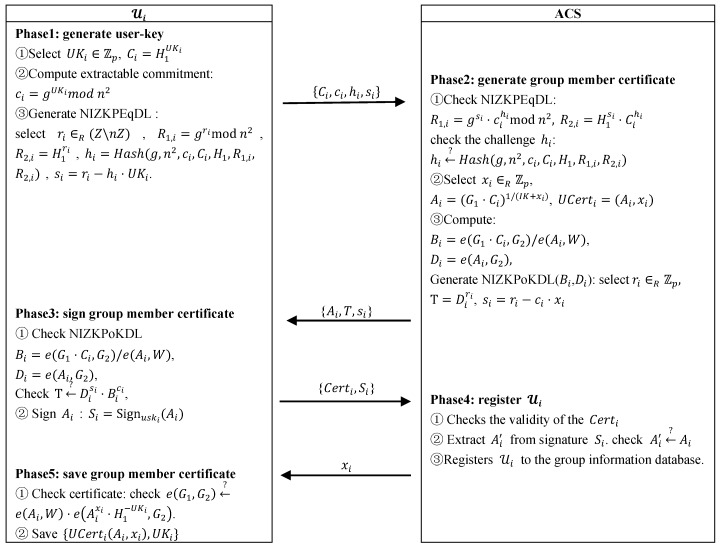
New user joining procedure.

Before joining a group, new users need to register with their real identity, assuming that each user has obtained a personal certificate and the associated public/private key pair [$upk_{i}$,$usk_{i}$] (in the PKI). When applying for joining a group, he has to prove to the ACS that he is a registered legitimate user in order to obtain his group member certificate and user-key. New users get the corresponding group public key from the blockchian according to the group they want to join, and perform the joining procedure as shown in Figure 3, where:

—The NIZKPEqDL is a zero-knowledge proof together with the extractable commitment becomes a proof of knowledge: the user $U_{i}$ know the user-key ${\mathrm{UK}}_{i}$.

—The NIZKPoKDL($B_{i}$,$D_{i}$) is a zero-knowledge proof of the discrete logarithm of $B_{i}$ in basis $D_{i}$. When receive the message, the user $U_{i}$ will check the NIZKPoKDL($B_{i}$,$D_{i}$) to confirm that the sender can issue the certificate.

The user $U_{i}$ generates the user-key $UK_{i}$, sending the knowledge proof NIZKPEqDL to the ACS. The ACS generates group member certificate $UCert_{i}=\left(A_{i},x_{i}\right)$ for $U_{i}$, and sends the knowledge proof NIZKPoKDL and the left half of $UCert_{i}$ namely $A_{i}$ to $U_{i}$. After $U_{i}$ verifies that the knowledge proof is valid, he signs $A_{i}$ with his private key $usk_{i}$ as $S_{i}={\mathrm{Sign}}_{usk_{i}}\left(A_{i}\right)$, then sends that and the personal certificate $Cert_{i}$ to the ACS. The ACS judge the validity of $Cert_{i}$, on success, it verifies the signature $S_{i}$ with the public key $upk_{i}$ of $Cert_{i}$. If the signature is valid, the ACS registers $U_{i}$ in the group information database, stores the signature, and sends the right half of the certificate $x_{i}$ to $U_{i}$. After verification, the user saves his group member certificate.

#### 4.2.3. Sign and Verify

After the user $U_{i}$ joins a group, he obtains the group member certificate and the user-key, and can generate group signatures to prove his group membership without revealing his personal identity. The procedure of generating and verifying the group signature is as follows:

$U_{i}$ generates the group signature:

1. Randomly chooses $\alpha_{n},\beta_{n}\in {\mathbb{Z}}_{p}$, then computes:
  (a) $T_{1,n}=K^{\alpha_{n}}$, $T_{2,n}=K^{\beta_{n}}$, $T_{3,n}=A_{i}\cdot H_{1}^{\alpha_{n}}\cdot H_{2}^{\beta_{n}}$.
2. Prove the knowledge of $\left\{\alpha_{n},\beta_{n},UCert_{i}\right\}$: randomly chooses $r_{\alpha ,n},r_{\beta ,n},r_{x,n},r_{y,n},r_{z,n}\in_{R}{\mathbb{Z}}_{p}$; computes:
  (a) $R_{1,n}=K^{r_{\alpha ,n}}$, $R_{2,n}=K^{r_{\beta ,n}}$, $R_{3,n}=e\left(T_{3,n}^{r_{x,n}}\cdot H_{1}^{-r_{z,n}}\cdot H_{2}^{-r_{y,n}},G_{2}\right)\cdot e\left(H_{1}^{-r_{\alpha ,n}},W\right)\cdot \;e\left(H_{2}^{-r_{\beta ,n}},W\right)$,
  (b) $c_{n}=Hash\left(M_{n},T_{1,n},T_{2,n},T_{3,n},R_{1,n},R_{2,n},R_{3,n}\right)$.
3. Set:
  (a) $s_{\alpha ,n}=r_{\alpha ,n}+c_{n}\cdot \alpha_{n}\mathrm{mod}\;p$, $s_{\beta ,n}=r_{\beta ,n}+c_{n}\cdot \beta_{n}\mathrm{mod}\;p$,
  (b) $s_{x,n}=r_{x,n}+c_{n}\cdot x_{i}\mathrm{mod}\;p$, $s_{y,n}=r_{y,n}+c_{n}\cdot y_{n}\mathrm{mod}\;p$,
  (c) $s_{z,n}=r_{z,n}+c_{n}\cdot z_{n}\mathrm{mod}\;p$ where $y_{n}=x_{i}\cdot \beta_{n}\mathrm{mod}\;p$, $z_{n}=x_{i}\cdot \alpha_{n}+UK_{i}\mathrm{mod}\;p$.
4. Obtain the group signature σ as $\left(T_{1,n},T_{2,n},T_{3,n},c_{n},s_{\alpha ,n},s_{\beta ,n},s_{x,n},s_{y,n},s_{z,n}\right)$.

ACS verifies the group signature:

1. Compute $R_{1,n}=K^{s_{a,n}}\cdot T_{1,n}^{-c_{n}}$, $R_{2,n}=K^{s_{\beta ,n}}\cdot T_{2,n}^{-c_{n}}$,
  (a) $R_{3,n}=e\left(T_{3,n}^{c_{n}}\cdot H_{1}^{-s_{\alpha ,n}}\cdot H_{2}^{-s_{\beta ,n}},W\right)\cdot e\left(T_{3,n}^{s_{x,n}}\cdot H_{1}^{-s_{z,n}}\cdot H_{2}^{-s_{y,n}}\cdot G_{1}^{-c_{n}},G_{2}\right)$.
2. Check $c_{n}\overset{?}{\leftarrow }Hash\left(M_{n},T_{1,n},T_{2,n},T_{3,n},R_{1,n},R_{2,n},R_{3,n}\right)$

#### 4.2.4. User Revokation

**Figure 4:**
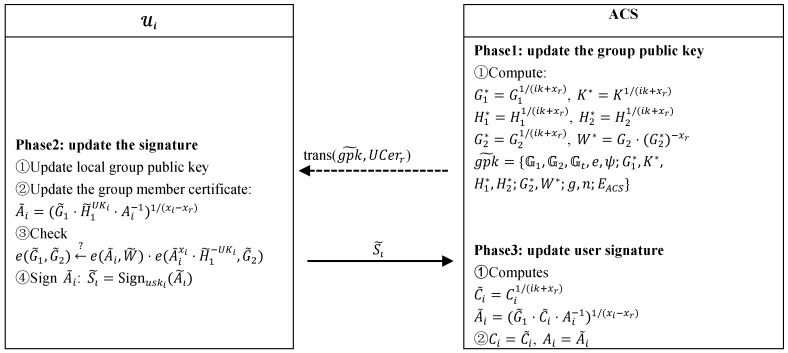
User revocation procedure.

User revocations occur when a user’s service subscriptions expire or behaviors violate network access policies. Instead of broadcasting revocation message to all unregistered users, the ACS invokes the smart contract to publish revocation transactions, which not only prevents the revocation message from being replayed or hijacked by the adversaries, but also reduces the revocation cost. Assume that a user with $UCer_{r}=\left(A_{r},x_{r}\right)$ is to be revoked. The revocation process is shown in Figure 4. The ACS updates the group public key and invokes the smart contract to publish the revocation transaction. After the unrevoked user $U_{i}$ listens to the revocation transaction, he updates the local group public key. Based on the updated group public key and revocation parameter $x_{r}$ in revocation transaction, $U_{i}$ calculates the new group member certificate ${\tilde{A}}_{i}$ and signs it with private key $usk_{i}$.Then $U_{i}$ sends the signature of ${\tilde{A}}_{i}$ to the ACS. After verifying that the signature is valid, the ACS updates the signature of $U_{i}$ in the group information database.

#### 4.2.5. Accountability and Disputes Resolution

**Figure 5:**
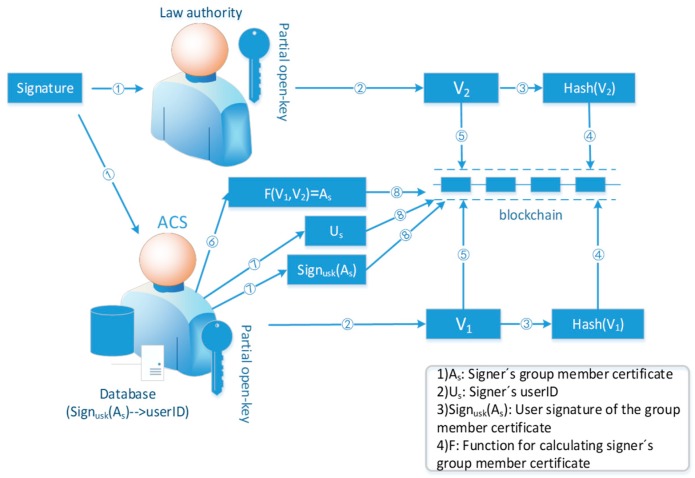
Accountability procedure in the proposed model.

If the user behavior violates the network access policy, the accountability phase is to be carried out. Different from the traditional accountability mechanism, this paper proposes a novel accountability mechanism based on blockchain as shown in Figure 5.

The scheme in this paper divides open-key into two parts, which are generated and saved by two different entities. To reveal the identity of the signer, two parts of the open-key are needed to participate in the calculation separately, and two intermediate results are used to calculate in the next step to get the group member certificate of the signer. To avoid cheating, the two arbitration entities need to submit a commitment to the blockchain first. By taking advantage of the transparent and irreversible properties of the blockchain, the two entities can’t modify their commitments after submitting them. After the both parties submit their commitments, they can disclose their respective calculation results. Then, the ACS calculates the group member certificate of the signer with the two results and announce it by the blockchain. Anyone can verify the correctness of the accountability result. 

Specifically, the accountability procedure is as follows:

1. The ACS calculates $V_{1}=T_{1,n}^{-\xi_{1}}$ = $H_{1}^{-\alpha_{n}}$ with the partial open-key $\xi_{1}$, and calculates the hash value $h\left(V_{1}\right)$ of $V_{1}$. Similarly, the LA calculates $V_{2}=T_{2,n}^{-\xi_{2}}=H_{2}^{-\beta_{n}}$ with the partial open-key $\xi_{2}$, and calculates the hash value $h\left(V_{2}\right)$ of $V_{2}$. Then they invoke the smart contract to publish the $h\left(V_{1}\right)$, $h\left(V_{2}\right)$ as commitments to the blockchain respectively.
2. When the ACS and the LA detect that both sides have submitted their commitments, they then invoke smart contract to submit respective calculation value $V_{1},\;V_{2}$. After that, the ACS computes group member certificates of signer $A_{s}=T_{3,n}\times V_{1}\times V_{2}$, and publishes it to the blockchain.
3. ACS finds the real identity and the corresponding signature $Sign_{usk}\left(A_{s}\right)$ of $A_{s}$ in the group information database to punish the misbehaving user.

### 4.3. Access Control Protocol

**Figure 6:**
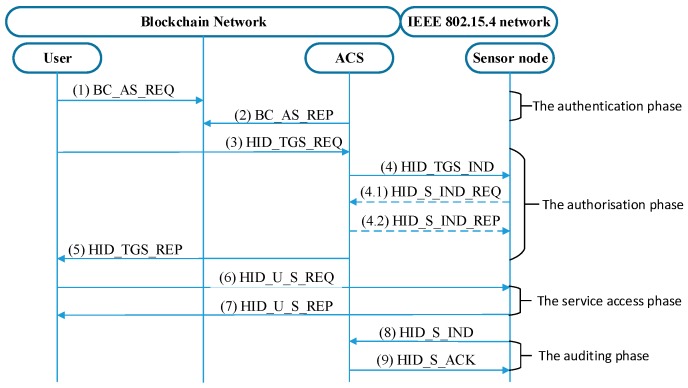
The enhanced Hidra architecture and message exchanges.

In this part, we introduce the access control protocol of our model. We enhance the Hidra protocol with unlinkability and anonymity of protocol messages. The enhanced Hidra protocol is shown in Figure 6 and Table 3 presents the terms used as abbreviations and the notation, while Table 4 records the specific content of each message. Each phase in the protocol is described in detail below.

#### 4.3.1. The Authentication Phase

In the authentication phase, the user $U_{i}$ generates group signature to authenticate himself against the ACS.AS. The AS can only check whether the requester is a member of the corresponding group, but not specialize which user in the group. If the above check succeeds, the AS issues the TGT to the requester through the blockchain. The operations at this phase are as following:

1. The user $U_{i}$ generates the one-time blockchain address (OTBA) $BCAddr_{U}$, chooses a radom $e_{U}\in {\mathbb{Z}}_{p}^{*}$ as the ECDH private key, and calculates the corresponding ECDH public key $E_{U}=e_{U}\times K$. $U_{i}$ generates request message $M_{n}$ including group identity $GID_{j}$, TGS identity $ID_{TGS}$, TGT validity term $Lifetime_{1}$, the OTBA $BCAddr_{U}$ and the ECDH public key $E_{U}$ used to establish a session key with the ACS.
2. Sign $M_{n}$ as group signature $\sigma_{n}$. Pack $\sigma_{n}$ and $M_{n}$ as a BC_AS_REQ transaction, destined for the ACS through the blockchain.

The ACS.AS keeps listening for any transactions destined to it. Once the AS finds a target transaction $Tans_{n}$, it extracts the group signature $\sigma_{n}$ and transfer it to the GSV for verification. On success, the AS generates a temporary identity $ID_{U}$ for the requester which is only valid within the TGT lifetime $Lifetime_{1}$, and store it in the active connections information database. The AS provides the requester with TGT, $ID_{U}$ and the instance of the key $K_{U,TGS}$ to communicate with the ACS.TGS by means of the BC_AS_REP message. One is encrypted with the $ESK_{U,ACS}=E_{U}\times e_{ACS}$.

#### 4.3.2. The Authorization Phase

After the previous phase, the user $U_{i}$ acquired TGT and his temporary identity $ID_{U}$, while no one can know his real identity, including the ACS. At this phase, users apply for service tickets by sending HID_TGS_REQ messages to ACS.TGS. In order to support the unlinkability of service access, two mechanisms are used to modify the HID_TGS_REP message which TGS responds to the requester, self-renewal TGT mechanism and fake ticket mechanism. Regarding the former, the TGS generates a new TGT for the requester. The updated TGT ticket is encrypted with the TGT Self-Renewal Key (TSRK), which is preset or randomly generated by the ACS. The new TGT ticket is embedded in a new type of padata field called PA-SR-TGT and carried in the PA-PRIV padata field of the HID_TGS_REP message. To implement the fake ticket mechanism, the original service ticket field is filled with invalid numbers, and the real service ticket is embedded in a new padata field called PA-TICKET, which is included in PA-PRIV padata. PA-PRIV provides integrity, confidentiality and anti-replay attacks, so the adversaries cannot establish any relationship with the subsequent message exchanges.

#### 4.3.3. The Service Access Phase and The Auditing Phase

After the user $U_{i}$ acquires the service ticket, he can initiate a service request to the RCD and send the service ticket to the target device through the message HID_U_R_REQ. After the device verifies that the service ticket is valid, it can be determined that the requester has been authenticated and authorized, and the message HID_U_S_REP is sent to respond to the requester to establish a security association. For further service requests, and local conditions specified in the related policy instance to make a local decision to grant access. In the subsequent service providing process, the device and the requester use session key Subkey in message HID_U_R_REQ to protect E2E data communication. In addition, each service access request will trigger the message HID_S_IND to send the access log (under which policies $ID_{Pol}$, who $ID_{U}$, at what time TIME, which services $ID_{R}$ and what actions $ID_{A}$ have been performed) to the ACS.ACM. After receive the message, the ACM associates the log with the requester’s group signature according to the requester’s temporary identity in the message, and stores the entry for recording, tracking, bookkeeping and further auditing purposes. After receiving the message HID_S_ACK returned by the ACM, the device deletes the log cache to prevent storage overflow.

### 4.4. Smart Contract Design

This section mainly introduces the relevant interface and algorithm logic in the smart contract used in the paper. Blockchain plays three main roles in the model: as a group public key management and publishing platform, as an intermediate platform for message exchanges between the ACS and users in the authentication phase, and opening accountability process to resolve potential disputes. Among them, the message exchanges does not require the smart contract, therefore the smart contract in our model implements two functions: group public key management and accountability process publicity. We design an smart contract GroupManager and three function interfaces in the contract:

IssueGroupPublicKey (groupID, groupPublicKey): This function can only be executed by the contract deployer, that is, the ACS. The ACS publishes the group public key to the blockchain as shown in Algorithm 1.

  **Algorithm 1** IssueGroupPublicKey  **Input:** groupID, groupPublicKey  **Output:** bool  1: **if** msg.sender is not AccessControlServer  2: **return** false;  3: **end if**  4: add groupID to Groups collection  5: mapping groupPublicKey to groupID  6: **return** true;

userRevoke(groupID, uCert, newGroupPublicKey): This function can only be executed by the ACS. When a user is revoked, the ACS publishes the group member certificate of the revoked user and the updated group public key to the blockchain as shown in Algorithm 2.

  **Algorithm 2** UserRevoke  **Input:** groupID, uCert, newGroupPublicKey  **Output:** bool  1: **if** msg.sender is not AccessControlServer **then**  2: **return** false;  3: **end if**  4: RevokedCert ← uCert  5: mapping newGroupPublicKey to groupID  6: **return** true;

Accountability (txid, signature, result/commitment, operating): This function can only be called by the ACS and the LA. As described in Algorithm 3, the corresponding operations include: createAccInst, submitCommitment, submitResult and submitSignerCert. To open a group signature, the ACS calls contract and perform the createAccInst operation to create an accountability instance and adds it to the accountability list AccList. The AccList is the mapping of blockchain transaction ID txid to the accountability instance. The parameter txid corresponds to the ID of the requesting transaction BC_AS_REQ where the group signature embedded needs to be accountable. The submitCommitment and the submitResult are performed to submit the commitment and median result respectively. The submitSignerCert can only be executed by the ACS to announce the signer’s group member certificate.

  **Algorithm 3** Accountability  **Input:** txid, signature, result/commitment, operating  **Output:** null  1: **if** operating is creatAccInst and msg.sender is ACS **then**  2: create and initialization newAccInst and  3: mapping newAccInst to txid: AccList[txid] ← signature, ACS_Commitment  4: **else if** operating is submitCommitment and msg.sender is LA **then**  5: AccList[txid] ← LA_commitment  6: **else if** operating is submitResult and msg.sender is ACS and HASH(ACS_result) equal to ACS_commitment **then**  7: AccList[txid] ← ACS_result  8: **else if** operating is submitResult and msg.sender is LawAuthorith and HASH(LA_result) equal to LA_commitment **then**  9: AccList[txid] ← LA_result  10: **else if** operating is submitSignerCert and msg.sender is ACS and LA_result, ACS_result have been assigned **then**  11: AccList[txid] ← signerCert  12: **end if**

## 5. Security Analysis

### 5.1. Resisting Privacy Attacks

In order to prevent the user privacy information from being analyzed and refined, it is necessary to ensure user anonymity and service access untraceability.

User anonymity is implemented by the group signature mechanism. The eavesdropper can only capture which group the authentication information is coming from, but not which member of the group. After authentication, the user obtains the temporary identity issued by the ACS and executes the subsequent protocol process with it. Temporary identity is encrypted in the whole process of the protocol so that it cannot be acquired by the adversaries. Therefore, in the later phase, the adversaries can neither know the identity of the requester nor which group the requester comes from. The temporary identity is valid only for the lifetime of the TGT ticket, and after that, the temporary identity entry will be deleted from the database.

To ensure the service access untraceability, it is necessary to achieve: (1) a user’s multiple requests at the same phase are unlinkable. (2) A user’s requests at different phase are unlinkable. For the first requirement, because the group signature scheme introduced in this model has the property of unlinkable, so that the two signatures generated by the same user cannot be linked. In the authorization phase, the self-renewed TGTs prevent the authorization request messages from being linked. For the second requirement, the ACS encrypts the requester’s temporary identity and the TGT ticket using the temporary session key based on ECDH in the authentication phase. Therefore, adversaries can’t get those information to associate the authentication request message with the authorization request message. In order to ensure the unlinkability between authorization request process and service access process, fake ticket mechanism is introduced in the authorization response message, real ticket is transported by PA-PRIV field which guarantees the freshness, confidentiality and integrity of the message. Therefore, the second goal can be implemented.

For the particularity of the role of the ACS (generates and issues the temporary identity for requesters), within a TGT lifetime, it can know which service requests come from the same user, but not the user’s identity.

### 5.2. Resisting to Replay Attacks

Users use the OTBA to send authentication request transactions, and the OTBA are embeded in messages that need to be signed. If an adversaries wants to replay the transaction, he must use the same blockchain address. According to the commonly used address generation algorithm of blockchain platform [38,39], it is computationally infeasible to replay messages by collision addresses. Every time users generate the authorization request, they increase $Nonce_{C,TGS}$ by i. If the value received by the ACS is lower than the expected value, the request message is considered invalid. If the value is valid, the ACS uses the $\left\{Nonce_{C,TGS}+i\right\}$ update database to resist the replay attack of HID_TGS_REQ message.

A one-way key mechanism is used to prevent message HID_TGS_IND from being replayed. One-way key function has the properties that forward computing (that is, to obtain $K^{L-1}$ given $K^{L}$) is easy and backward computing(that is, to obtain $K^{L+1}$ given $K^{L}$ ) is unfeasible. Before sending a message HID_TGS_IND to the node for the first time, ACS will preset a key chain with a length of N [$K_{s,cm}^{1}$…$K_{S,CM}^{N}$]. Each subsequent message HID_TGS_IND is embedded in the next value of the key chain in sequence. According to the properties of one-way function, if a node calculates the function value $F\left(K_{S,TGS}^{i}\right)$ corresponding to the embedded $K_{S,TGS}^{i}$ in the message equal to the last value $K_{S,TGS}^{i-1}$ of the key chain stored locally, it can confirm that the message is fresh. The first time a node receives a message HID_TGS_IND, it needs to exchange a pair of messages HID_S_IND_REQ/REP with ACS to get the value $K_{S,TGS}^{i-1}$ for validation. In HID_U_S_REQ message, the service ticket itself is disposable, so it can resist replay attack.

### 5.3. Resisting to Resource Consumption Attacks

The ACS is a centralized device, so it is vulnerable to resource attacks. An adversary can send a large number of messages or requests to it in order to consume its resources [40]. In the authentication phase, if adversaries access the ACS on a large scale simultaneously by generating valid or invalid signatures, resource consumption attacks will occur. Blockchains are used as an intermediate platform for message exchanges between users and the ACS. Due to the fully distributed property of blockchain, attacks on individual nodes will not affect the whole blockchain network, which can effectively resist resource consumption attacks.

In the authorization phase of the protocol, a legitimate user can use one or more valid IDs to initiate a large number of request messages, consuming ACS resources. In this phase, the server can limit the maximum number of requests per user by setting a threshold of $Nonce_{C,TGS}$ to prevent users from launching resource consumption attacks.

### 5.4. Guarantee the Openness and Transparency of the Accountability Process

**Figure 7:**
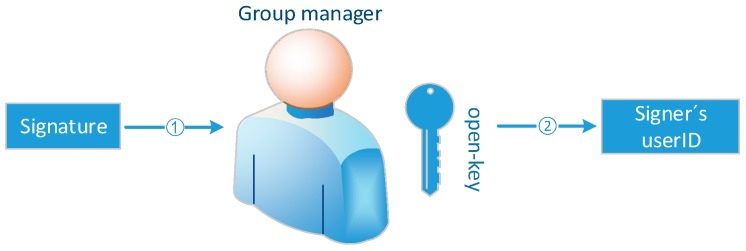
Traditional accountability procedure.

**Figure 8:**
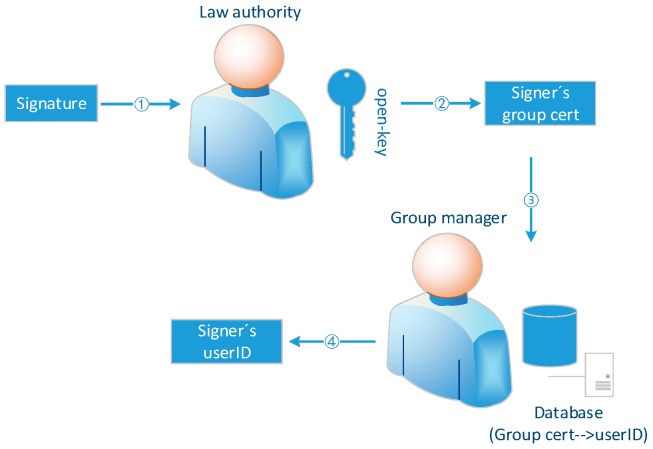
Accountability procedure in APAC model.

In the accountability phase, the traditional method is that the entity holding full open-key gives the identity of the signer after calculation, as shown in Figure 7. In the APAC model, open-key and user real identity are stored separately on two different entities: LA and group administrator. After the group member certificate of the signer is calculated by the LA, the group administrator queries the real user identity corresponding to the group member certificate in the database, as shown in Figure 8. In these two accountability mechanisms, only the calculation results given by the entities holding open-key can be seen from outside, but the accountability process can’t be witnessed. AS publishing the accountability process is equivalent to publish the open-key, the privacy security of the access control system will collapse. In the case that entity holding open-key is semi-trusted, the result is not convincing. There is the possibility that the entity holding open-key colludes with the signer (or it is the signer itself) to blame other users. The accountability mechanism in this paper divides open-key into two parts generated and saved by two entities. The two arbitration entities are mutually reinforcing and cannot unilaterally influence the accountability result. In the process of opening group signature, both arbitration entities need to submit a commitment to the blockchain before submitting their own median value, so that the latter submitter cannot designing its own calculation value to influent the accountability result based on the value of other entity. By taking advantage of transparent and irreversible properties of the blockchain, the two entities can’t modify their commitments after submitting them. Assuming that the hash function used in our model is secure enough, the both entities can’t extract each other’s calculation results from the commitment, so that they can’t design the results to frame others. What’s more, anyone can witness the accountability process and verify its correctness. Additionally, because the signer uses the private key representing his true identity to sign the group member certificate, it can also guarantee the non-repudiation of the signature.

## 6. Performance Evaluation

Extensions proposed in the model inevitably incur additional costs. In this section, we analyze the performance of the privacy-preserving model and the feasibility of the enhanced Hidra protocol of eHAPAC.

### 6.1. Performance Evaluation of the Enhanced Hidra Protocol

**Figure 9:**
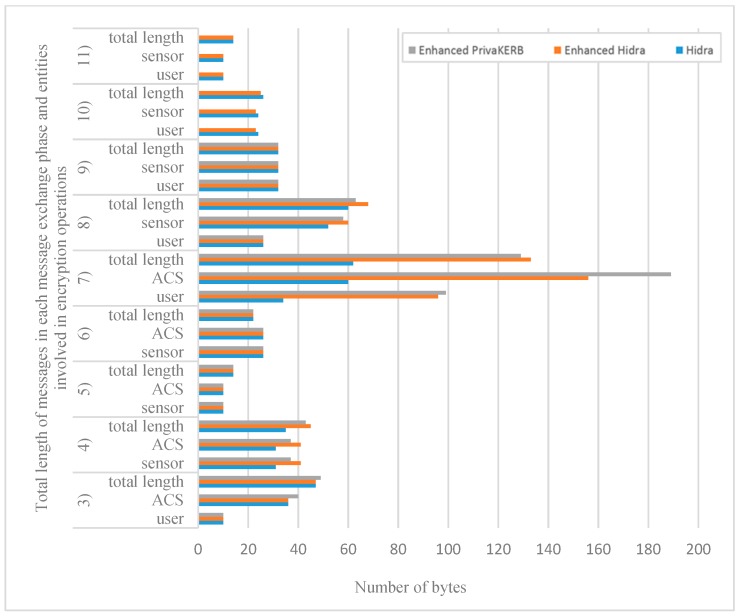
Comparison among enhanced PrivaKERB protocol, Hidra protocol, and enhanced Hidra protocol in the total length of messages and the number of bytes over which each entity must perform cryptographic operations.

The enhanced Hidra protocol extends the original Hidra protocol with a self-renewal TGT mechanism and fake ticket mechanism referring to paper [20]. From the perspective of protocol performance evaluation, it only increases the length of messages received, sent and the number of bytes to perform cryptographic operations. In Table 5, we collect the lengths of messages defined in the enhanced Hidra protocol and the number of bytes that each entity needs to encrypt/decrypted in each phase. The authentication request message involves the calculation of group signatures, the cost of this part will be analyzed later. Figure 9 shows the comparison among the enhanced PrivaKERB [20], the Hidra protocol [24] and the enhanced Hidra protocol in the number of bytes over which each entity must perform cryptographic operations (including symmetric encryption operations and generating message authentication code(MAC)) and the total length of messages in each message exchange phase. Among them, the enhanced PrivaKERB [20] takes the value in Level 3, because the privacy-preserving requirements of the Level 3 are similar to those of this paper. Additionally, the Hidra and enhanced PrivaKERB have been validated in C0 RDCs. As can be seen from the Figure 9, for message 3), 5), 6), 9), 10), 11), the number of bytes to perform cryptographic operations on each entity in our enhanced Hidra protocol is less than or equal to the Hidra and the enhanced PrivaKERB. For message 7), the number of bytes to perform cryptographic operations on each entity in our protocol is longer than that in the Hidra, but less than that in the enhanced PrivaKERB. For message 4) and 8), the number of bytes needed to be encrypted in our protocol is slightly longer than that of the other two protocols. In Message 4), our protocol only has two bytes more symmetric encryption and two bytes more MAC operations than the Hidra. For Message 8), our protocol has two bytes more symmetrical encryption operations in the ACS side and the sensors side than that in the enhanced PrivaKERB. The above analysis indicates that the increase in protocol message length and cryptographic operations by our extension of the original Hidra protocol is very small.

**Figure 10:**
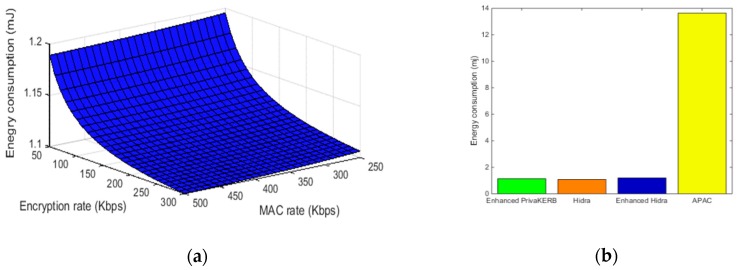
Energy consumption results: (**a**) impact of the MAC computation rate and the encryption rate on the energy consumption of sensor nodes; (**b**) energy consumption comparison of sensor nodes among enhanced Hidra, enhanced PrivaKERB and Hidra.

Similar to [19,41,42] we estimated the computational energy consumption (E) in the sensor node by the formula E=U∗I∗ t, where U is the voltage in volts (V), I is the current of the circuit in milliamps (mA), and t is execution time (s). For measuring purposes, the values of U and I are derived from the Crossbow data sheet of TelosB. The TelosB is a common sensor platform that has an 8 MHz CPU, 10 KB RAM and 48KB ROM. In active mode of TelosB, U = 3 V and I = 1.8 mA. The execution time t is calculated by the formula:

$$t=\frac{L_{CRYP}}{R_{CRYP}}$$

where $L_{CRYP}$ is the length of encrypted field, and the $R_{CRYP}$ is the cryptographic operations rate. The cryptographic operations rate can vary significantly hinging on specific cryptographic algorithm used, the optimization method used and specific hardware platform [43]. Like [20], We have evaluated the performance for a broad range of cryptographic operations execution rates, from pessimistic values to optimistic values. On the other hand, regarding the communication energy consumption, we have evaluated based on the energy model from Meulenaer et al. [44]. Receiving and transmitting a single bit of data on TelosB costs 0.81 μJ and 0.72 μJ respectively. Figure 10a,b shows the energy consumption assessment results on the sensor nodes for establishing a secure session. The impact of encryption rate and MAC computing rate on energy consumption in our protocol is depicted in Figure 10a. As can be seen from the figure, the energy computation of sensors even in the worst case, with the lowest MAC and encryption computation rate, is below 1.2 mJ. In the sensor node powered by AA alkaline batteries, it can establish a secure connection of about ten million times. The figure also shows that improving the encryption rate has a greater impact on the reduction of the energy computation than does improving the MAC computation rate. Figure 10b shows the energy consumption comparison of sensor nodes among the enhanced Hidra protocol, the enhanced PrivaKERB protocol, the original Hidra protocol and the APAC protocol in the worst case. Compared with the basic Hidra protocol and the enhanced PrivaKERB protocol, our protocol enhances security but only adds minimal energy consumption. Additionally, the energy consumption of our protocol much less than the APAC protocol because the APAC performs group signature verification on sensors, but our protocol transfers computing burden to servers and clients. It’s easy to say that our approach is a good choice to implement access control privacy-preserving in resource-constrained environments.

In terms of storage, our model does not request implementing complex security mechanisms on sensor nodes, only involves symmetric encryption and message authentication code (MAC) calculation. The symmetric-key algorithm widely used in WSNs such as AES, require no more than 2KB RAM, 9KB ROM in TelosB. The widely used MAC algorithms such SHA-1 require no more than 1KB RAM, 6.6KB ROM in TelosB referring to [45]. Additionally, the permanent storage required by the protocol to store the key, protocol code and temporary log is much smaller than the storage capacity that the RCD can provide. Assuming that a sensor platform uses the Contiki operating system (typical configuration requires 2 KB RAM, 40 KB ROM) [46], the storage requirements of the enhanced Hidra can still be met and therefore the enhanced Hidra can satisfies the feasibility of most severely C0 RCDs (less than 10KB of data and less than 100 KB of code).

We tested the performance of symmetric encryption and MAC algorithms on Raspberry Pi, the common hardware platform of the IoT. We choose the Raspberry Pi 3 Model B as the test platform to run the 128-bit AES and SHA-1 MAC algorithms. As can be seen from Table 6, the time consumed by encryption is extremely small. Even if the length of encryption is 130 bytes, the time consumed on encryption is no more than 1 millisecond, while in our protocol the number of bytes needed to be encrypted on sensor nodes is within 60 at a time.

### 6.2. Performance Evaluation of the Privacy-Preserving Model

**Figure 11:**
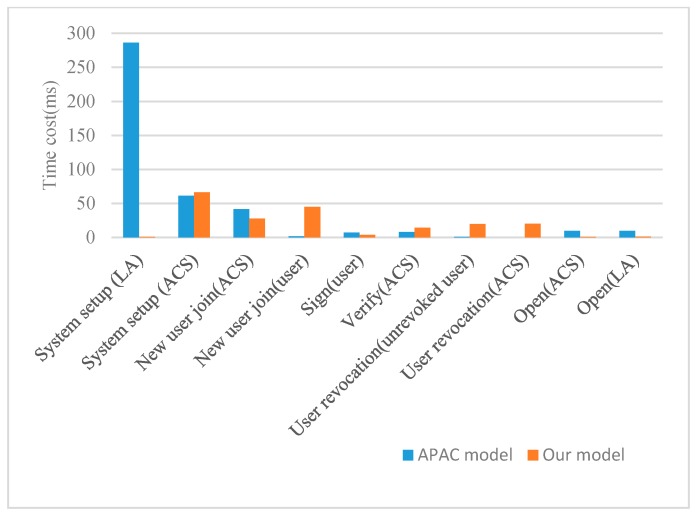
Comparison between the APAC model and our privacy-preserving framework in the time cost.

Introducing group signatures increases computational cost on the three entities of the model: the LA, the ACS and the users, but does not increase any overhead on the RCDs. We use C language (OpenSSL, PBC, GMP) to implement the calculation process of the privacy-preserving model, in which the pairing type uses the D type pairing defined by the PBC library. RSA key length is set to 1024 bits, which is considered secure enough for now and immediate future. The SHA-3 hash algorithm (Keccak 256) is chosen to use in the group signature scheme implementation, which has 256 hash bit length. The testing process is executed on personal PC (with 2-GB RAM) in Ubuntu 11.04 environment. We test the time consumption of each phase of our privacy-preserving model at different CPU frequencies, and show the results in Table 7 and Table 8. Some phases have several sub-processes, and the time consumption of each sub-process is also given in the table, separated by “/”. We compare the results measured at CPU frequency of 2.0 GHz with the reference model APAC, as shown in Figure 11.

As can be seen from the graph, in the system setup phase, the time cost in the LA side of the APAC model is much more than that in our privacy-preserving model. When the number of user groups in the system is large, our model can greatly improve the efficiency of the system setup phase. However, the time cost of our model is higher than that of the APAC model at user side in the new user joining phase, and at both ACS side and user side in the user revocation phase. This increase in time cost is due to the fact that the ACS needs to store the user’s signature of group member certificate so that the user can possess certificate in an undeniable way to ensure non-repudiation of accountability results. The user revocation phase is also the same. Unrevoked users need to re-sign group member certificates, and the ACS need to verify and replace the certificate signatures of unrevoked users, so the computational overhead is slightly increased in these two phases. However, in order to ensure the non-repudiation of the accountability result, we believe that the introduction of computational overhead is worthwhile.

## 7. Conclusions

The key challenges for secure remote access to IP-enabled RCDs are the availability of feasible access control solutions and preserving user data access privacy. This paper establishes a privacy-preserving access control model eHAPAC for IP-enabled WSNs, a severely resourced-constrained environment. This paper enhances the formally validated Hidra access protocol with unlinkability of message exchanges. In the authorization phase, the self-renewed TGTs is used to prevent the authorization request messages from being linked. A fake ticket mechanism is introduced in the authorization response message to ensure the unlinkability between authorization request process and service access process. This paper improves the group signature-based APAC privacy-preserving access control model by setting up two mutually restrictive third parties to avoid third-party monitoring and cheating. The proposed model ensures user data access privacy without disclosure to any entity including the third parties participating in the security protocol, which makes it more practical. This paper modifies the XSGS group signature scheme and chooses it as an example to describe the implementation process of the enhanced privacy-preserving model. However, there are still some problems: it is unable to resist resource attacks, the accountability process of group signatures cannot be above board, which easily causes disputes, and the management and publishing methods of group public keys are not flexible enough. To this end, this paper introduces blockchain technology and designs a smart contract to solve these problems taking advantage of the distributed, transparent and irreversible attributes of the blockchain. In the system setup phase, blockchain acts as a platform for group public key management and publishing to increase the flexibility of public key management. In the authentication phase, blockchain serves as an intermediate platform for message exchange between ACS and users to resist resource consumption attacks on ACS. In the accountability phase, this paper proposes a new accountability mechanism based on blockchain, which makes the accountability process to prevent arbitration organizations from cheating, and makes the results more convincing. The security analysis shows that the proposed model can meet our expected security goals. Through experimental simulations and analyzing the performance of the proposed model, it is demonstrates that the proposed model is feasible and rationality.

## Figures and Tables

**Table 1:** Comparison among Hidra, Ladon and Kerberos.

	Kerberos	Ladon	Hidra
Targeted protected devices	Powerful workstations	Severely resource deprived devices	Severely resource deprived devices
Authentication and key establishment	√	√	√
Authorization		√	√
Independence of clock synchronization		√	√
Dynamic fine-grained policy enforcement			√
Accurate accounting			√

**Table 2:** Terminology and notation agreement of the privacy-preserving model of eHAPAC.

Expression	Description
$U_{i}$	a registered user who requests the sensor services
IK	issue-key, used to issue group member certificates
OK	open-key, consisting of two parts which generated by ACS and LA respectively
$e_{X}$	ECDH private key of entity X
$E_{X}$	ECDH public key of entity X
gsk	group private key
gpk	group private key
${\mathrm{UK}}_{i}$	user-key, used to generate group signature
$UCert_{i}$	group member certificate
$Cert_{i}$	personal certificate
$upk_{i}$	public key of $Cert_{i}$
$usk_{i}$	private key of $Cert_{i}$

**Table 3:** Terminology and Notation agreement.

Expression	Description
U	Registered user
BC	Blockchain
AS	Authentication server
TGS	Ticket granting server
S	Sensor node
σ	Group signature
$GID_{j}$	Group identity
$BCAddr_{X}$	Blockchain address of entity X
$E_{X}$	ECDH public key of entity X, used to establish session key with the communication partner
$ESK_{X,Y}$	Temporary session key based on ECDH between X and Y
$K_{X,Y}$	Secret key shared between entities X and Y
$K_{X}$	Secret key of entity X shared with the ACS
$K_{X,Y}^{i}$	i-th value of a one-way key chain used to provide freshness in the communication between entities X and Y
Subkey	Session key shared between the user and the target sensor node

**Table 4:** Details of the content of enhanced Hidra messages.

Message	Direction	Content
BC_AS_REQ	U→BC	M||σ *M*={$GID_{j}\left|\left|ID_{TGS}\right|\right|Lifetime_{1}\left|\left|BCAddr_{U}\right|\right|E_{U}$}
BC_AS_REP	AS→BC	$GID_{j}||\left\{Ticket_{TGS}\left|\left|K_{U,TGS}\right|\right|Nonce_{U,TGS}\left|\left|ID_{TGS}\right|\right|ID_{U}\right\}ESK_{U,ACS}$ $Ticket_{TGS}=\left\{K_{U,TGS}\left|\left|ID_{U}\right|\right|Nonce_{U,TGS}\right\}K_{TGS}$
HID_TGS_REQ	U→TGS	$ID_{S}\left|\left|lifetime_{2}\right|\right|Nonce_{1}\left|\left|Ticket_{TGS}\right|\right|AuthN_{TGS}$ $Ticket_{TGS}=\left\{K_{U,TGS}\left|\left|ID_{U}\right|\right|Nonce_{U,TGS}\right\}K_{TGS}$ $AuthN_{TGS}=\{ID_{U}||Nonce_{U,TGS}+i\}K_{U,TGS}$
HID_TGS_IND	TGS→S	$ID_{S}\left|\left|anon@anon\right|\right|Nonce_{U,S}\left|\left|Lifetime_{2}\right|\right|K_{S,TGS}^{i}\left|\left|AuthZ\right|\right|PA-PRIV||MAC$ $\mathrm{AuthZ}=\left\{Policy_{R}\right\}K_{R}$ $PA-PRIV=\{Nonce_{U,S}||ID_{U}\}K_{S}$ $MAC=\left\{K_{S},ID_{U}\left|\left|Nonce_{U,S}\right|\right|Lifetime_{2}\left|\left|K_{S,TGS}^{i}\right|\right|AuthZ\right\}$
HID_S_IND_REQ	S→TGS	$ID_{S}\left|\left|Nonce_{2}\right|\right|MAC(K_{S},ID_{S}||Nonce_{2})$
HID_S_IND_REP	TGS→S	$ID_{S}\left|\left|K_{S,TGS}^{i+1}\right|\right|MAC\left(K_{S},ID_{S}\left|\left|Nonce_{2}\right|\right|K_{S,TGS}^{i+1}\right)$
HID_TGS_REP	TGS→U	$anon@anon\left|\left|Fake-Tickets_{S}\;\right|\right|\{K_{U,S}\left|\left|Nonce_{U,S}\right|\right|Nonce_{1}||ID_{S}\}K_{U,TGS}||$ PA−PRIV $Fake-Ticket_{S}=\{Flag_{F}\;||invalid-data\}$ $PA-PRIV=\left\{Nonce_{1}\left|\left|PA-SR-TGT\;\right|\right|PA-TICKET\right\}K_{U,TGS}$ $PA-SR-TGT=\left\{K_{U,TGS}\left|\left|ID_{U}\right|\right|Nonce_{U,TGS}\right\}K_{TGS}$ PA−TICKET*=*{$K_{U,S}\left|\left|ID_{U}\right|\right|Nonce_{U,S}\left|\left|Attr_{G}\right|\right|Attr_{C}$}$K_{S}$
HID_U_S_REQ	U→S	$Ticket_{S}\left|\left|AuthN_{S}\right|\right|Nonce_{3}$ $Ticket_{S}=\left\{K_{U,S}\left|\left|ID_{U}\right|\right|Nonce_{U,S}\left|\left|Attr_{G}\right|\right|Attr_{C}\right\}K_{S}$ $AuthN_{S}=\left\{ID_{U}\left|\left|Nonce_{U,S}\right|\right|Subkey\right\}K_{U,S}$
HID_U_S_REP	S→U	$\left\{Nonce_{U,S}\left|\left|Subkey\right|\right|Nonce_{3}\right\}K_{U,S}$
HID_S_IND	S→ACM	$ID_{S}||\left\{Nonce_{4}\left|\left|ID_{Pol}\right|\right|Log_{U,S}\right\}K_{S}$ $Log_{U,S}=\left\{ID_{U}\left|\left|ID_{R}\right|\right|ID_{A}\left|\left|TIME\right|\right|Nonce_{5}+i\right\}$
HID_S_ACK	ACM→S	$ID_{S}\left|\left|Nonce_{4}\right|\right|MAC(K_{S},ID_{S}||Nonce_{4})$

**Table 5:** Lengths of THE enhanced Hidra protocol messages and number of bytes over which each entity must perform cryptographic operations.

Message Type	Length (Bytes)	Bytes Subject to Cryptographic Operations				
		User (Bytes)	AS (Bytes)	TGS (Bytes)	ACM (Bytea)	Sensor (Bytes)
1)BC_AS_REQ	308	71	71	-	-	-
2)BC_AS_REP	56	54	82	-	-	-
3)HID_TGS_REQ	47	10	-	36	-	-
4)HID_TGS_IND	45	-	-	41	-	41
5)HID_TGS_IND_REQ	14	-	-	10	-	10
6)HID_S_IND_REP	22	-	-	26	-	26
7)HID_TGS_REP	133	96	-	156	-	-
8)HID_U_S_REQ	68	26	-	-	-	60
9)HID_U_S_REP	32	32	-	-	-	32
10)HID_S_IND	25	-	-	-	23	23
11)HID_S_ACK	14	-	-	-	10	10

**Table 6:** Execution time of AES and SHA-1 on Raspberry Pi 3B.

Item	Value			
Length of the plaintext (byte)	10	50	90	130
AES encryption Time (ms)	0.5213	0.5220	0.5249	0.5261
AES decryption Time (ms)	0.5471	0.5492	0.5508	0.5529
SHA-1 encryption Time (ms)	0.1360	0.1452	0.1611	0.1713

**Table 7:** Running time for some phases of our privacy-preserving framework.

	System Setup (ACS)	System Setup (LA)	New User Joining (ACS: Two Phases)	New User Joining (User: Three Phases)
Time (CPU = 1.6 GHz) (ms)	74.536	1.325	33.525/0.258	7.192/27.375/21.022
Time (CPU = 1.8 GHz) (ms)	73.456	1.105	29.693/0.231	6.372/24.579/18.694
Time (CPU = 2.0 GHz) (ms)	66.214	1.000	27.358/0.206	5.798/22.028/16.921
Time (CPU = 2.2 GHz) (ms)	63.311	0.939	24.988/0.195	5.516/20.853/15.989
Time (CPU = 2.4 GHz) (ms)	50.464	0.808	23.983/0.189	5.062/19.397/14.688
Time (CPU = 2.6 GHz) (ms)	44.418	0.756	21.654/0.170	4.650/17.818/13.696
Time (CPU = 2.8 GHz) (ms)	43.204	0.728	20.300/0.166	3.978/15.272/11.773
Time (CPU = 3.1 GHz) (ms)	41.501	0.616	18.390/0.147	3.804/14.496/11.111

**Table 8:** Running time for the remaining phases of our privacy-preserving framework.

	Sign (User)	Signature Verify (ACS)	User Revocation (User)	User Revocation (ACS: Two Phase)	Open (ACS: Two Phase)	Open (LA)
Time (CPU = 1.6 GHz) (ms)	4.516	18.321	27.538	22.876/2.854	1.246/0.010	1.207
Time (CPU = 1.8 GHz) (ms)	4.015	15.819	23.852	19.397/2.657	1.097/0.010	1.116
Time (CPU = 2.0 GHz) (ms)	3.694	14.421	19.568	18.001/2.312	1.033/0.008	0.990
Time (CPU = 2.2 GHz) (ms)	3.230	13.148	17.512	15.939/2.078	0.930/0.010	0.875
Time (CPU = 2.4 GHz) (ms)	3.052	12.120	16.248	14.502/1.883	0.768/0.007	0.766
Time (CPU = 2.6 GHz) (ms)	2.949	11.978	14.994	13.523/1.777	0.701/0.006	0.689
Time (CPU = 2.8 GHz) (ms)	2.652	10.711	13.879	12.376/1.637	0.653/0.005	0.647
Time (CPU = 3.1 GHz) (ms)	2.435	9.503	13.404	11.413/1.492	0.598/0.005	0.604
